# Oral CoQ10 attenuates high salt-induced hypertension by restoring neurotransmitters and cytokines in the hypothalamic paraventricular nucleus

**DOI:** 10.1038/srep30301

**Published:** 2016-07-25

**Authors:** Hong-Li Gao, Xiao-Jing Yu, Jie Qi, Qiu-Yue Yi, Wang-Hui Jing, Wen-Yan Sun, Wei Cui, Jian-Jun Mu, Zu-Yi Yuan, Xiu-Fang Zhao, Kai-Li Liu, Guo-Qing Zhu, Xiao-Lian Shi, Jin-Jun Liu, Yu-Ming Kang

**Affiliations:** 1 Department of Physiology and Pathophysiology, School of Basic Medical Sciences, Cardiovascular Research Center, Xi’an Jiaotong University Health Science Center, Xi’an 710061, China; 2 Department of Pharmaceutical Analysis, School of Pharmacy, Xi’an Jiaotong University Health Science Center, Xi’an 710061, China; 3 Department of Endocrinology and Metabolism, First Affiliated Hospital of Xi’an Jiaotong University, Xi’an Jiaotong University Health Science Center, Xi’an 710061, China; 4 Department of Cardiology, First Affiliated Hospital of Medical College of Xi’an Jiaotong University, Xi’an 710061, China; 5 Department of Physiology, Nanjing Medical University, Nanjing 210029, China; 6 Department of Pharmacology, School of Basic Medical Sciences, Xi’an Jiaotong University Health Science Center, Xi’an 710061, China

## Abstract

High salt intake leads to an increase in some proinflammatory cytokines and neurotransmitters involved in the pathogenesis of hypertension. The purpose of this work was to know if oral administration of anti-oxidant and free-radical scavenger CoQ10 may attenuate high salt-induced hypertension via regulating neurotransmitters and cytokines in the hypothalamic paraventricular nucleus (PVN). Adult male Sprague-Dawley (SD) rats were fed with a normal salt diet (NS, 0.3% NaCl) or a high salt diet (HS, 8% NaCl) for 15 weeks to induce hypertension. These rats received CoQ10 (10 mg/kg/day) dissolved in olive oil was given by gavage (10 mg/kg/day) for 15 weeks. HS resulted in higher mean arterial pressure (MAP) and the sympathetic nerve activity (RSNA). These HS rats had higher PVN levels of norepinephrine (NE), tyrosine hydroxylase (TH), interleukin (IL)-1β, NOX2 and NOX4, lower PVN levels of gamma-aminobutyric acid (GABA), IL-10, copper/zinc superoxide dismutase (Cu/Zn-SOD) and the 67-kDa isoform of glutamate decarboxylase (GAD67), as compared with NS group. CoQ10 supplementation reduced NE, TH, IL-1β, NOX2 and NOX4 in the PVN, and induced IL-10, Cu/Zn-SOD and GAD67 in the PVN. These findings suggest that CoQ10 supplementation restores neurotransmitters and cytokines in the PVN, thereby attenuating high salt-induced hypertension.

Hypertension, one of the major cardiovascular diseases in the world, has become a serious public health problem affecting one-third of adults globally, which also makes a heavy burden on our society today^1^. Epidemiology clinical trials have shown that the more sodium people intake daily, the higher the blood pressure people will get^2^,^3^,^4^. High salt intake induces imbalance between pro- and anti-inflammatory cytokines and neurotransmitters in the hypothalamic paraventricular nucleus (PVN) with the increase in blood pressure or sympathetic activity^5^.

The PVN plays a crucial role in the development of hypertension and is recognized as an important central site for the coordination and regulation of autonomic response^6^,^7^,^8^. A growing body of evidences support that high salt intake increases proinflammation cytokines (PICs), such as tumour necrosis factor-alpha (TNF-α), interleukin-1β (IL-1β), and the IL-6, decreased anti-inflammatory cytokines (AICs) such as IL-10, in the PVN which have been associated with a serious cardiovascular risk in hypertension^8^,^9^,^10^,^11^. The PVN is a site where a number of neurotransmitters, excitatory as well as inhibitory, converge to influence the sympathetic nervous activity. Glutamate and norepinephrine (NE) (a marker of sympathetic activity) are excitatory neurotransmitters, and gamma-aminobutyric acid (GABA) is a dominant inhibitory neurotransmitter, which play an important role in inducing sympathetic response in the PVN. Previous studies in our group found that hypertensive rats had increased higher levels of glutamate, NE and lower level of GABA in the PVN^12^.

CoQ10 is an anti-oxidant and free-radical scavenger which is like vitamins C, vitamins E and glutathione against oxidative stress^13^,^14^,^15^. It has been applied for a potential supplementary treatment of hypertension^16^,^17^,^18^, including high salt-induced hypertension. However, CoQ10 its antioxidant properties in the PVN of hypertension have not been elucidated and it is unknown whether CoQ10 supplementation attenuates hypertensive response via modulating PICs and neurotransmitters in the PVN. Therefore, the present study was aimed to determine: (i) whether CoQ10 supplementation attenuates salt-induced hypertension; (ii) whether CoQ10 exerts protective effects on hypertension via restoring the balance between pro- and anti-inflammatory cytokines, and the excitatory and inhibitory neurotransmitters in the PVN.

## Materials and Methods

### Animals

Healthy male adult Sprague-Dawley rats weighing 250 g~270 g were used in this study. All of the rats were housed in a room maintained under controlled light (12 h/12 h dark/light cycle) and temperature (20–23 °C) conditions. All of the experimental procedures were approved by the Xi’an Jiaotong University Committee for Animal Research and were in accordance with the National Institutes of Health Guide for the Care and Use of Laboratory Animals.

### General experimental protocol

The NS group received a normal salt (NS, 0.3% NaCl) diet and the HS group received a high salt (HS, 8% NaCl) diet. The SD rats were randomly divided into four groups: (i) NS + oil; (ii) NS + CoQ10; (iii) HS + oil; (IV) HS + CoQ10. CoQ10 (Sigma St. Louis, MO) was dissolved in olive oil and was given daily at a dose of 10 mg/kg/day via oral gavage for 15 weeks^19^,^20^. Control olive oil group was given daily with olive oil (0.5 ml/kg/day) via oral gavage for 15 weeks. At the end of 15 weeks, rats were anesthetized for the sympathetic nerve activity (RSNA) measurement and euthanized later to collect samples for molecular and immunofluorescence studies.

### Measurement of mean arterial pressure (MAP)

Blood pressure was determined by a tail-cuff occlusion and acute experiment method. as described previously^21^. To achieve the steady pulse, rats were allowed to habituate to this procedure for 3 days prior to each experiment. Unanesthetized rats were warmed to an ambient temperature of 38 °C by placing rats in a holding device mounted on a thermostatically controlled warming plate. Blood pressure values were averaged fromsix consecutive cycles per week obtained from each rat.

At the end of the15 week, rats were anesthetized with a ketamine (80 mg/kg) and xylazine (10 mg/kg) mixture intra-peritoneally (ip). The femoral artery was cannulated with polyethylene catheters prior filled with 0.1 ml heparinized saline (50 units/ml) and connected to a pressure transducer (MLT0380, ADInstruments, Australia) for continuous MAP recording. MAP data was collected for 30 min and averaged.

### Electrophysiological recording

After 15 weeks of blood pressure recorded, the rats were anaesthetized with a ketamine (80 mg/kg) and xylazine (10 mg/kg) mixture (ip) for recording RSNA. Methods for recordings and integrating RSNA have been described previously^7^,^22^.

### Collection of tissue samples

Rats were decapitated when still under anesthesia. Tissue samples were stored at −80 °C until assayed. The PVN tissue was isolated following Palkovits’s microdissection procedure as previously described^23^. The tissues were collected from both sides of the PVN of individual rat. Tissue samples were stored at −80 °C until assayed^24^.

### Immunohistochemical and immunofluorescence studies

The primary antibodies for NOX2 (sc-5827), NOX4 (sc-21860), IL-1β (sc-1251), IL-10 (sc-1783), GAD67 (sc-7512) and TH (sc-14007) were purchased from Santa Cruz Biotechnology. The methods for PVN immunohistochemical and immunofluorescence staining were conducted as previously described^8^. For each rat, the positive neurons for NOX2, NOX4, IL-1β, IL-10, TH and GAD67 within the bilateral borders of the PVN were manually counted in three consecutive sections and reported as an average value.

### Dihydroethidium staining

Superoxide generation in the PVN was determined by fluorescent-labeled dihydroethidium (DHE; Molecular Probes). DHE staining was performed as previously described^7^,^25^.

### Detemination of neurotransmitters in PVN by high-performance liquid chromatography

High-performance liquid chromatography (HPLC) with electrochemical detection (Waters-2465, Waters Corporation, USA) was used for measuring norepinephrine, glutamate and GABA in PVN as previously described^24^,^26^,^27^. Briefly, samples or standards were derivatized with o-phthaldialdehyde; 20 μl of the resulting mixture was automatically loaded onto column using a refrigerated autoinjector. The columns used for NE, glutamate and GABA were purchased from Waters, (Ireland), including Symmetry^®^ C18 column (4.6 × 150 mm, 5.0 μm), Symmetry Shield^TM^ RP18 column (4.6 × 150 mm, 5.0 μm) and Xbridge BEH Amide column (3.0 × 100 mm, 2.5 μm) respectively. The mobile phase consisted of NaH_2_PO_4_ (0.05 M, pH 6.8) with 20% methanol, and the flow rate was 1 ml/min delivered by a Waters pump. NE, glutamate and GABA concentration were detected and analyzed using Empower 3 analytical software (Waters).

### Western blot

The tissue homogenate proteins were extracted from the PVN, The primary antibodies for NOX2 (sc-5827), NOX4 (sc-21860), antioxidant enzymes superoxide dismutase (Cu-Zn superoxide dismutase-1, SOD-1) (sc-11407), IL-1β (sc-1251), IL-10 (sc-1783), GAD67 (sc-7512), and TH (sc-14007) were purchased from Santa Cruz Biotechnology. Protein loading was controlled by probing all western blots with anti-β-actin antibody (Santa Cruz Biotechnology). The procedures of western blot were described previously^28^,^29^. Protein detection was performed using the enhanced chemiluminescence kit using ChemiDoc XRS System (Bio-rad, USA). The bands were analyzed by NIH Image J software.

### Statistical analysis

All data are expressed as mean ± SE. MAP data were analyzed by repeated-measures ANOVA. Other data were analyzed by Tukey’s test. Results were considered significant when a probability value of *P* < 0.05.

## Result

### Effects of CoQ10 on MAP in salt-induced hypertensive rats

**Figure 1:**
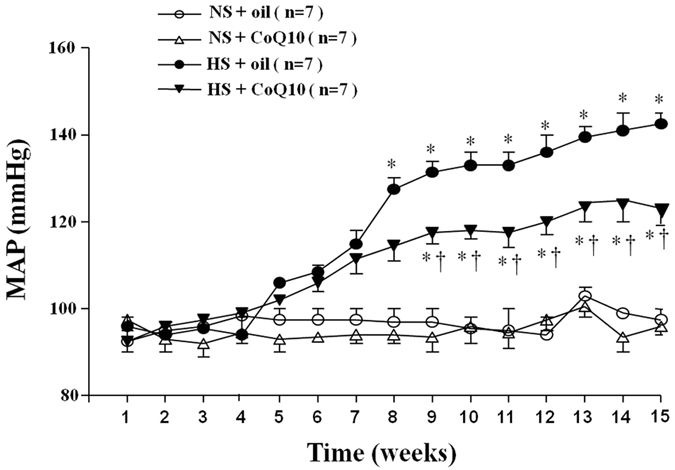
Effects of CoQ10 on mean arterial pressure (MAP) in salt-induced hypertensive rats. An 8% NaCl diet has been used in high salt (HS) diet rats. MAP was increased gradually after HS diet compared with NS diet groups (*P* < 0.05, n = 7). When compared with HS + oil rats, 15-week of CoQ10 administration by gavage at the dose of 10 mg/kg attenuated high salt diet-induced hypertension (*P* < 0.05, n = 7). Values are expressed as means ± SE. *P < 0.05 vs control (NS + oil or NS + CoQ10), ^†^*P* < 0.05, HS + CoQ10 vs HS + oil.

An 8% NaCl diet has often been used in HS diet studies in rats. To assess the effect of CoQ10 on hypertensive response in salt-induced hypertension, MAP was monitored by using a noninvasive computerized tail cuff system. As shown in Fig. 1, the baseline blood pressure among all rats was at the same level. Blood pressure of rat receiving high salt diet was incresed gradually since 4^th^ week of high salt diet. At 8^th^ week of high salt diet, HS rats showed a significant increase in MAP when compared with NS group and maintain at high level until up to the end of experiment. CoQ10 administration by gavage at the dose of 10 mg/kg/day attenuated high salt-induced hypertension compared with model group.

### Effects of CoQ10 on renal sympathetic nerve activity (RSNA) in high salt-induced hypertensive rats

**Figure 2:**
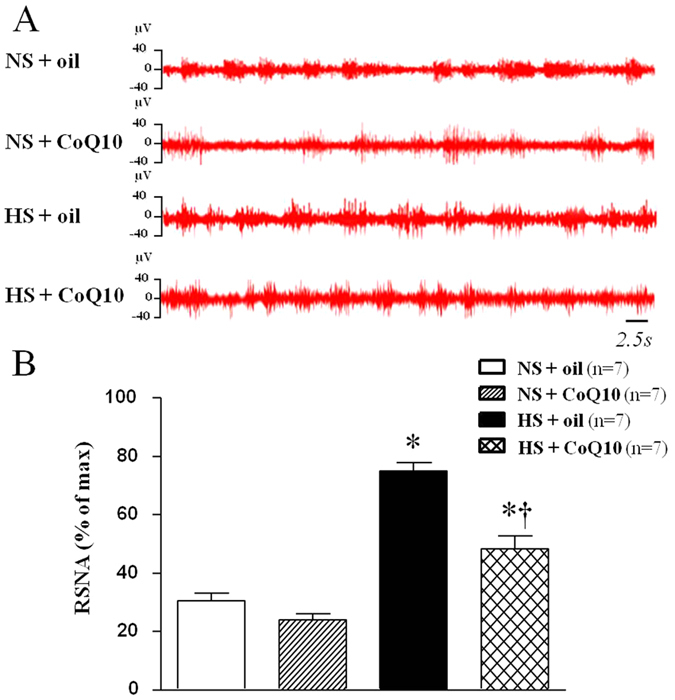
Effects of CoQ10 on renal sympathetic nerve activity (RSNA) in high salt-induced hypertensive rats. Results of electrophysiological technology for RSNA showed that HS rats exhibited a significant higher RSNA (% of max) compared with the NS rats (*P* < 0.05, n = 7). When compared with HS + oil rats, 15-week of CoQ10 administration by gavage at the dose of 10 mg/kg attenuated RSNA (*P* < 0.05, n = 7). (**A**) Representative renal sympathetic nerve activity in different groups. (**B**) Bar graph comparing renal sympathetic nerve activity in different groups. Values are expressed as means ± SE. *P < 0.05 vs control (NS + oil or NS + CoQ10), ^†^*P* < 0.05, HS + CoQ10 vs HS + oil.

Renal sympathetic nerve activity is an important direct indicator for the evaluation of sympathetic central activity and was measured by electrophysiological method at the end of experiment. As shown in Fig. 2, HS rats exhibited a significant higher RSNA (% of max) compared with the NS group. Fifteen weeks of CoQ10 administration by gavage at the dose of 10 mg/kg/day attenuated RSNA which is consisted with changes of blood pressure. Increased RSNA can contribute to the genesis of hypertension. It suggests that CoQ10 inhibited the elevated sympathetic activity in salt induced hypertension.

### Effects of CoQ10 on oxidative stress in the PVN of high salt-induced hypertensive rats

**Figure 3:**
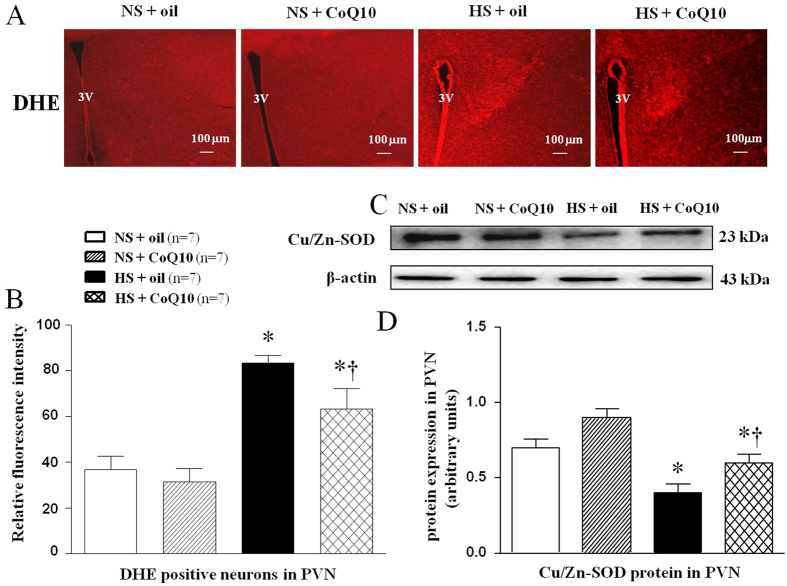
Effects of CoQ10 on PVN levels of the superoxide and Cu/Zn-SOD proteins expression in the PVN of high salt-induced hypertensive rats. ROS activity was measured by fluorescent-labeled dihydroethidium (DHE) staing and Cu/Zn-SOD proteins were measured by Western blot. Results of showed that HS rats had stronger fluorescence intensity labeled with DHE and lower Cu/Zn-SOD level compared with NS rats (*P* < 0.05, n = 7). 15-week of CoQ10 administration by gavage at the dose of 10 mg/kg/day significantly decreased DHE staining and increased Cu/Zn-SOD protein expression (*P* < 0.05, n = 7). (**A**) A representative immunofluorescence image of DHE. (**B**) Densitometric analysis of immunofluorescent intensity of DHE in the PVN in different groups. (**C**) A representative immunoblot of Cu/Zn-SOD. (**D**) Densitometric analysis of protein expression of Cu/Zn-SOD in the PVN in different groups. Values are expressed as means ± SE. *P < 0.05 vs control (NS + oil or NS + CoQ10), ^†^*P* < 0.05, HS + CoQ10 vs HS + oil, 3V, third ventricle.

Superoxide anion levels in PVN were determined by fluorescent-labeled dihydroethidium (DHE) staining. To testify the reason of excessive oxidative stress, Cu/Zn-SOD proteins expression was meassured by Western blot. As shown in Fig. 3, HS rats had stronger fluorescence intensity of DHE (Fig. 3A,B), and lower SOD level (Fig. 3C,D) in PVN compared with NS rats. CoQ10 administration by gavage at the dose of 10 mg/kg/day significantly decreased DHE staining and increased Cu/Zn-SOD proteins expression in PVN. It suggests that CoQ10 attenuated oxidative stress through strenghing scavenging activity of SOD in salt-induced hypertensive rats.

**Figure 4:**
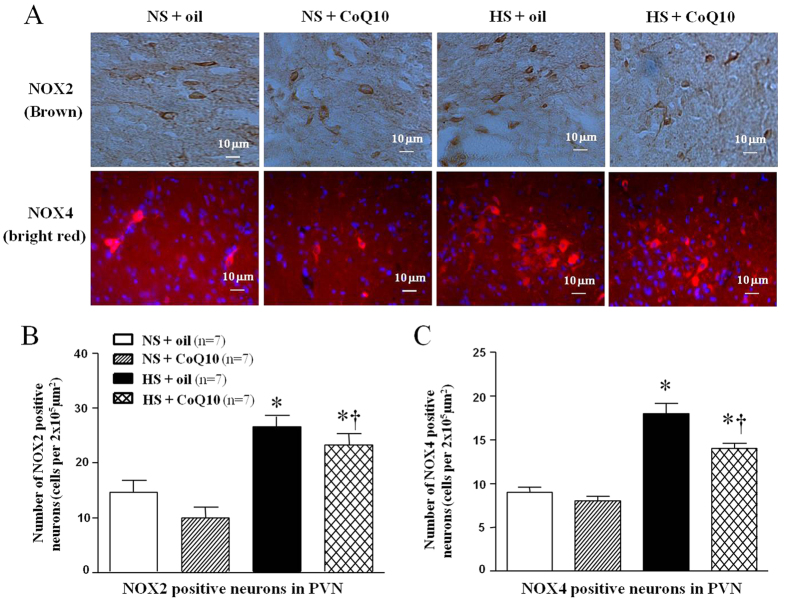
Effects of CoQ10 on NAD (**P**) H oxidase subunit NOX2 and NOX4 (membrane- associated oxidase proteins) in the PVN of high salt-induced hypertensive rats by immunohistochemical and immunofluorescence staining. Results showed that HS rats had stronger fluorescence intensity of NOX2 and NOX4 compared with NS rats (*P* < 0.05, n = 7). 15-week of CoQ10 administration by gavage at the dose of 10 mg/kg/day significantly decreased NOX2 and NOX4 expression (*P* < 0.05, n = 7). (**A**) A representative immunohistochemical staining of NOX2 and a representative immunofluorescence staining of NOX4. (**B**) Densitometric analysis of immunofluorescent intensity of NOX2 in the PVN in different groups. (**C**) Densitometric analysis of immunofluorescent intensity of NOX4 in the PVN in different groups. Values are expressed as means ± SE. *P < 0.05 vs control (NS + oil or NS + CoQ10), ^†^*P* < 0.05, HS + CoQ10 vs HS + oil.

**Figure 5:**
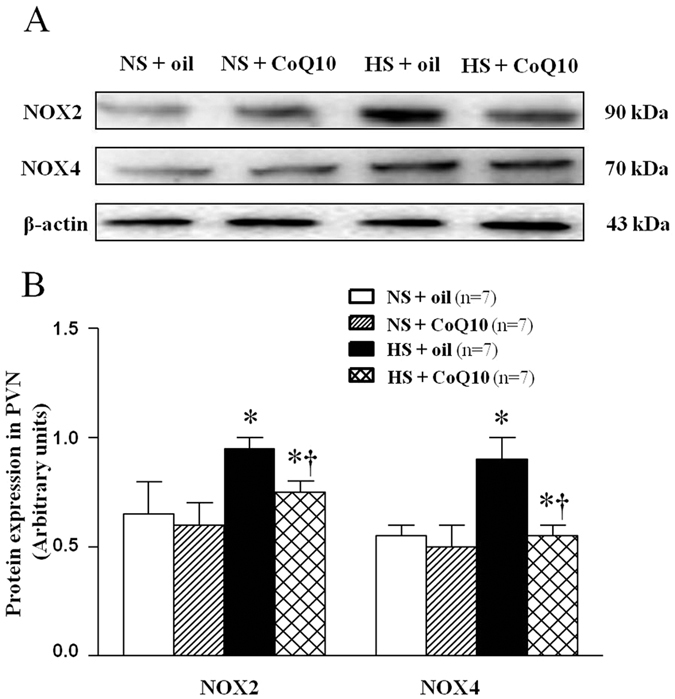
Effects of CoQ10 on NAD (**P**) H oxidase subunit NOX2 (a membrane- associated oxidase protein) and NOX4 in the PVN of high salt-induced hypertensive rats by Western blot. Results showed that HS rats had higher protein expression of NOX2 and NOX4 compared with NS rats (*P* < 0.05, n = 7). 15-week of CoQ10 administration by gavage at the dose of 10 mg/kg/day significantly decreased NOX2 and NOX4 expression (*P* < 0.05, n = 7). (**A**) Representative immunoblot of NOX2 and NOX4. (**B**) Densitometric analysis of protein expression of NOX2. Effects of CoQ10 on NAD (P) H oxidase subunit NOX2 and NOX4 in the PVN in different groups. Values are expressed as means ± SE. *P < 0.05 vs control (NS + oil or NS + CoQ10), ^†^*P* < 0.05, HS + CoQ10 vs HS + oil.

The major source of ROS is the nonphagocytic NAD(P)H oxidase, which is composed of membrane- associated gp91^*phox*^ (also known as NOX2) and p22^*phox*^ (also known as NOX4), as well as the cytosolic subunits such as p47^*phox*^, p67^*phox*^, and the small GTPase Rac^30^. To observe the origin of superoxide anion in PVN, immunohistochemical and immunoflurescence staining and Western blot were used to measure NAD (P) H oxidase subunits, NOX2 and NOX4, in PVN. As shown in Fig. 4, high-salt diet increased both NOX2 (Fig. 4A,B) and NOX4 (Fig. 4A,C) immunoflurescence intensity which means the increase of amount of NOX2 and NOX4 positive neurons. CoQ10 administration by gavage at the dose of 10 mg/kg/day significantly decreased the expression of NOX2 and NOX4 expression in the PVN, compared with NS group. For Western blot result, we observed the same changes which mean CoQ10 also attenuated protein expression of NOX2 (Fig. 5) and NOX4 (Fig. 5) in PVN of hypertensive rats.

### Effects of CoQ10 on the levels of norepinephrine (NE), glutamate and gamma-aminobutyric acid (GABA) in the PVN of high salt-induced hypertensive rats

**Figure 6:**
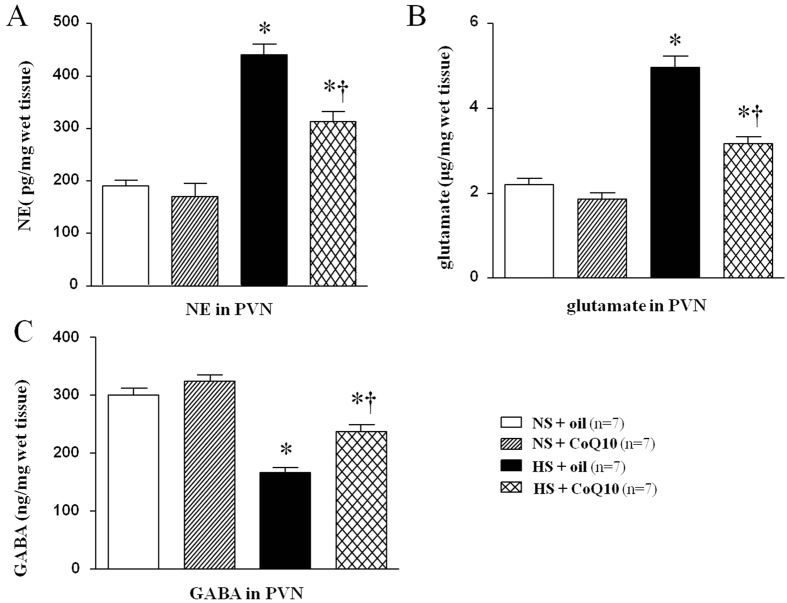
Effects of CoQ10 on the levels of norepinephrine (NE), glutamate and increased GABA in the PVN of high salt-induced hypertensive rats. These neurotransmitters were assayed by HPLC. HS rats had higher levels of NE (**A**) and glutamate (**B**), and lower levels of GABA (**C**) in the PVN compared with NS rats (*P* < 0.05, n = 7). 15-week of CoQ10 administration by gavage at the dose of 10 mg/kg significantly decreased the PVN levels of NE, glutamate and increased GABA (*P* < 0.05, n = 7). *P < 0.05 vs control (NS + oil or NS + CoQ10), ^†^*P* < 0.05, HS + CoQ10 vs HS + oil.

Glutamate and NE are excitatory neurotransmitters while GABA is a dominant inhibitory neurotransmitter. We measured these neurotransmitters in PVN by HPLC. As shown in Fig. 6, HS rats had higher level of NE (Fig. 6A) and glutamate (Fig. 6B) in the plasma when compared with normal saline rats, but lower level of GABA in PVN (Fig. 6C). These results suggest unbalance between excitatory and inhibitory neurotransmitters in PVN of salt-induced hypertensive rats. 15-week of CoQ10 administration by gavage at the dose of 10 mg/kg/day significantly decreased the levels of NE and glutamate, and increased the level of GABA in the PVN, compared with the control groups. It suggests that CoQ10 equilibrate excitatory and inhibitory neurotransmitters in PVN in salt-induced hypertensive rats.

### Effects of CoQ10 on TH and GAD67 levels in the PVN of high salt-induced hypertensive rats

**Figure 7:**
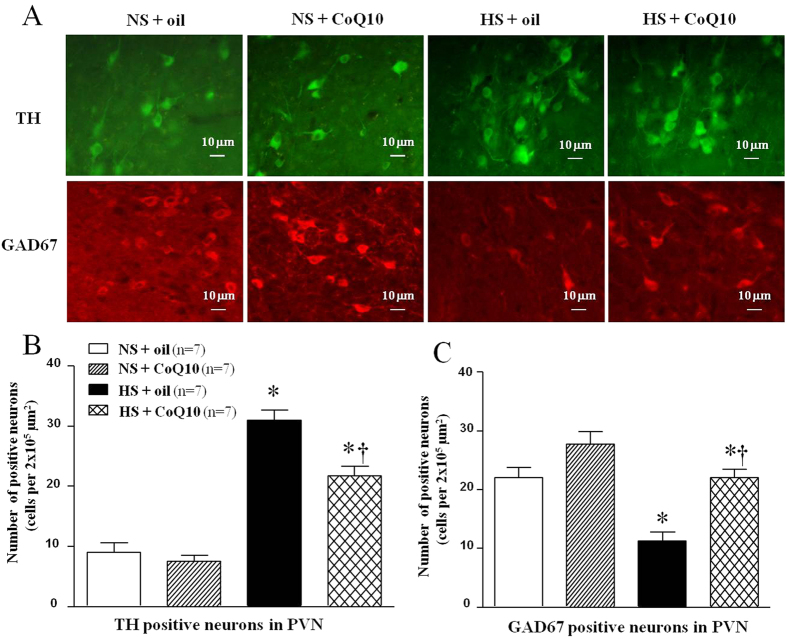
Effects of CoQ10 on the levels of TH and GAD67 in the PVN of high salt-induced hypertensive rats by immunofluorescence staining. Results showed that HS rats had higher levels of TH and lower levels of GAD67, compared with the NS rats (*P* < 0.05, n = 7). 15-week of CoQ10 administration by gavage at the dose of 10 mg/kg reduced TH and increased GAD67, compared with NS rats (*P* < 0.05, n = 7). (**A**) Representative immunofluorescence staining of TH and GAD67. (**B**) Densitometric analysis of immunofluorescent intensity of TH. (**C**) Densitometric analysis of immunofluorescent intensity of GAD67 in the PVN in different groups. Values are expressed as means ± SE. *P < 0.05 vs control (NS + oil or NS + CoQ10), ^†^*P* < 0.05, HS + CoQ10 vs HS + oil.

**Figure 8:**
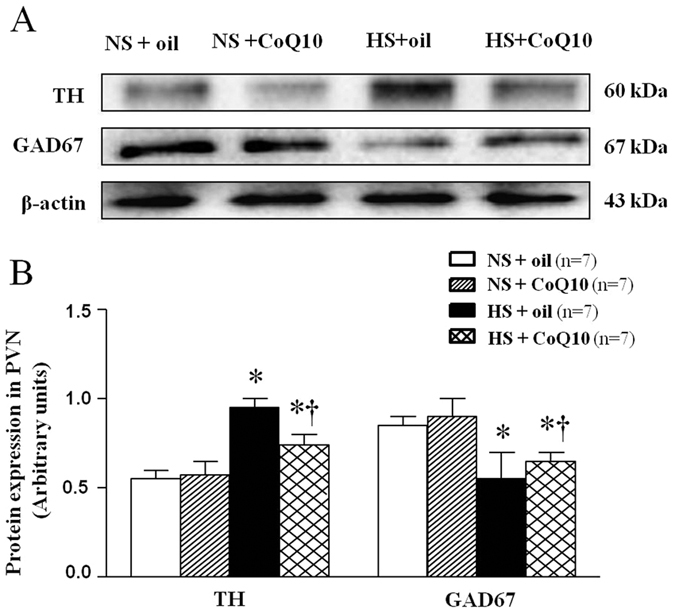
Effects of CoQ10 on the levels of TH and GAD67 in the PVN of high salt-induced hypertensive rats by Western blot. Results showed that HS rats had higher levels of TH and lower levels of GAD67, compared with the NS rats (*P* < 0.05, n = 7). 15-week of CoQ10 administration by gavage at the dose of 10 mg/kg reduced TH and increased the GAD67 level compared with NS rats (*P* < 0.05, n = 7). (**A**) Representative immunoblot of TH and GAD67. (**B**) Densitometric analysis of protein expression of TH and GAD67 in the PVN in different groups. Values are expressed as means ± SE. *P < 0.05 vs control (NS + oil or NS + CoQ10), ^†^*P* < 0.05, HS + CoQ10 vs HS + oil.

Immunoflurescence staining and Western blot were used to measure tyrosine hydroxylase (TH) and the 67-kDa isoform of glutamate decarboxylase (GAD67) levels in the PVN. TH and GAD are related to the synthesis of neurotransmitters, NE and GABA respectively. As shown in results of immunoflurescence staining, HS rats showed higher levels of TH (Fig. 7A,B) in the PVN compared with the NS group. 15-week of CoQ10 administration by gavage at the dose of 10 mg/kg/day reduced the levels of TH in the PVN, compared with control rats. Brain L-glutamate decarboxylase (GAD) is the enzyme responsible for the synthesis of GABA. This result is coincident with PVN level of GABA. As shown in results of immunoflurescence staining, HS rats showed lower levels of GAD67 (Fig. 7A,C) in the PVN compared with the NS group. 15-week of CoQ10 administration by gavage at the dose of 10 mg/kg/day increased the levels of GAD67 in the PVN, compared with control rats. Tyrosine hydroxylase catalyzes the rate limiting step in synthesis of noradrenaline in the central nervous system. This result is coincident with PVN level of NE. Samilar results were observed when protein expression of TH and GAD67 were determined by Western blot. It suggests that CoQ10 also attenuated protein expression of TH (Fig. 8) and increased the protein expression of GAD67 (Fig. 8) in PVN of hypertensive rats. Taken together, studies on neurotransmitters and their related enzymes confirmed that CoQ10 equilibrate excitatory and inhibitory neurotransmitters in PVN in salt-induced hypertensive rats.

### Effects of CoQ10 on IL-1β and IL-10 levels in the PVN of high salt-induced hypertensive rats

**Figure 9:**
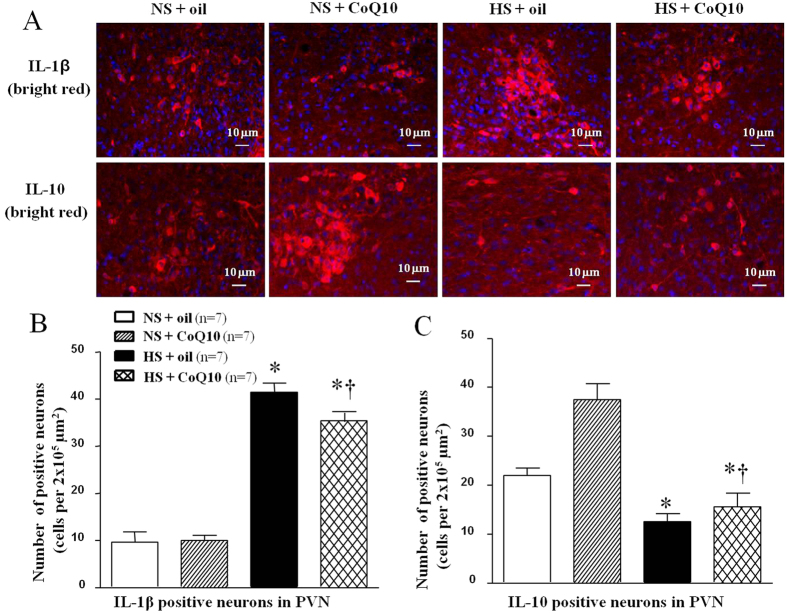
Effects of CoQ10 on the level of IL-1β and IL-10 in the PVN of high salt-induced hypertensive rats by immunofluorescence staining. Results showed that HS increased the level of IL-1β but decreased the level of IL-10 (*P* < 0.05, n = 7). 15-week of CoQ10 administration by gavage at the dose of 10 mg/kg significantly decreased the expression of IL-1β but increased the level of IL-10, compared with the NS rats (*P* < 0.05, n = 7). (**A**) Representative immunofluorescence staining of IL-1β and IL-10. (**B**) Densitometric analysis of immunofluorescent intensity of IL-1β. (**C**) Densitometric analysis of immunofluorescent intensity of IL-10 in the PVN in different groups. Values are expressed as means ± SE. *P < 0.05 vs control (NS + oil or NS + CoQ10), ^†^*P* < 0.05, HS + CoQ10 vs HS + oil.

**Figure 10:**
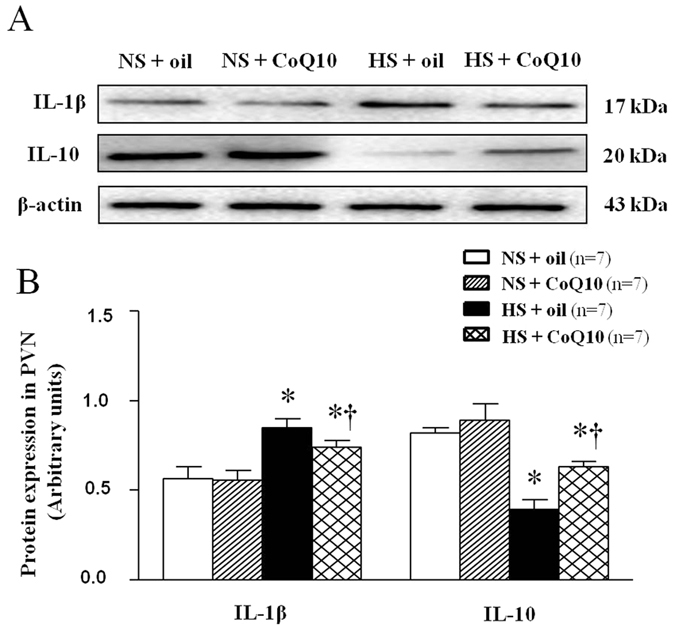
Effects of CoQ10 on the level of IL-1β and IL-10 in the PVN of high salt-induced hypertensive rats by Western blot. Results showed that HS increased the level of IL-1β and decreased the level of IL-10 (*P* < 0.05, n = 7). 15-week of CoQ10 administration by gavage at the dose of 10 mg/kg significantly decreased the expression of IL-1β and increased the level of IL-10 compared with the NS rats (*P* < 0.05, n = 7). (**A**) Representative immunoblot of IL-1β and IL-10. (**B**) Densitometric analysis of protein expression of IL-1β and IL-10 in the PVN in different groups. Values are expressed as means ± SE. *P < 0.05 vs control (NS + oil or NS + CoQ10), ^†^*P* < 0.05, HS + CoQ10 vs HS + oil.

IL-1β is one of the proinflammation cytokines while IL-10 is an anti-inflammatory cytokine. Both IL-1β and IL-10 in PVN were evaluated by immunoflurescence staining and Western blot. As shown in Fig. 9, high-salt diet increased IL-1β expression (Fig. 9A,B), but inhibited IL-10 expression in PVN (Fig. 9A,C). 15-week of CoQ10 administration by gavage at the dose of 10 mg/kg/day significantly decreased the expression of IL-1β, and increased the expression of IL-10 in the PVN, compared with the NS group. For Western blot result, we observed the same changes which mean CoQ10 also attenuated protein expression of IL-1β (Fig. 10) and IL-10 (Fig. 10) in PVN of hypertensive rats.

### Effects of CoQ10 on the serum IL-1β and IL-10 in high salt-induced hypertensive rats

**Figure 11:**
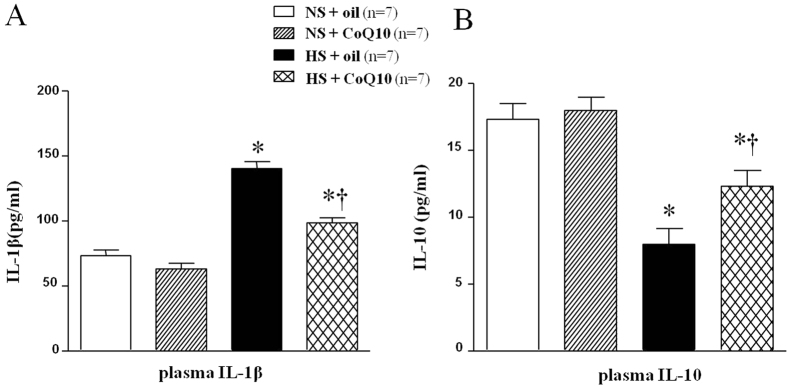
Effects of CoQ10 on the serum IL-1β and IL-10 in high salt-induced hypertensive rats. ELISA results showed that HS diet rats had higher level of serum IL-1β (**A**) while lower level of IL-10 (**B**). 15-week of CoQ10 administration by gavage at the dose of 10 mg/kg significantly decreased the expression of serum IL-1β and increased the level of IL-10 compared with the NS rats (*P* < 0.05, n = 7). Values are expressed as means ± SE. *P < 0.05 vs control (NS + oil or NS + CoQ10), ^†^*P* < 0.05, HS + CoQ10 vs HS + oil.

Serum IL-1β and IL-10 were assayed by ELISA. As shown in Fig. 11, HS diet rats had higher level of serum IL-1β (Fig. 11A) and lower level of IL-10 (Fig. 11B). 15-week of CoQ10 administration by gavage at the dose of 10 mg/kg significantly decreased the expression of serum IL-1β and increased the level of IL-10 compared with the NS rats.

## Discussion

The novel finding in this study is that long-term administration of CoQ10 attenuates MAP and RSNA by restoring the balance between pro- and anti-inflammatory cytokines and the balance between excitatory and inhibitory neurotransmitters in the PVN.

In this study, we demonstrated that high salt diet increase IL-1β and decrease IL-10 in the PVN, and CoQ10 supplement could down-regulate the level of IL-1β and increase the level of IL-10. Recent studies suggest that PVN infusion of gevokizumab (IL-1β inhibitor) attenuates hypertensive responses by restoring the balance between pro- and anti-inflammatory cytokines and reducing the oxidative stress in the PVN of salt-sensitive hypertension rats^5^. Furthermore, pentoxifylline (TNF-α blocker) within the PVN causes decreased arterial pressure and cardiac hypertrophy in ANG II-induced hypertension^31^. Other study suggest that CoQ10 exerts its anti-inflammatory effects by gene expression modification reducing the activity of inflammatory markers in RAW 264.7 expressing human apoE3 or apoE4^32^. Similarly, CoQ10 has also been observed to inhibit the expression of IL-6, TNF-α and NF-κB, an anti-inflammatory effect mediated via gene expression modification or antioxidant/radical-scavenging activity in Rheumatoid Arthritis Patients^33^. Consistent with these findings, this study demonstrated the therapeutic effect of long-term administration of CoQ10 supplement attenuated high salt-induced hypertension by restoring the balance between pro- and anti-inflammatory cytokines in the PVN. We also assessed the circulating levels of IL-1β and IL-10. Consistent with PVN results, serum IL-1β was higher and serum IL-10 was lower and CoQ10 supplement inhibited serum IL-1β and increased serum IL-10. Since the rats took high salt diet, both systemic and local effect participate in the production of these cytokines.

Recent studies have shown that oxidative stress in the PVN emerged as major player in sympathoexcitation of salt-sensitive hypertension^11^,^34^. NADPH oxidase-derived ROS is increased in the various types of hypertension^12^,^22^,^35^. We found that levels of NAD (P) H subunits (NOX2 and NOX4) in the PVN in HS group were significantly increased and expression of antioxidant enzymes superoxide dismutase (Cu-Zn superoxide dismutase-1, SOD-1) decreased, as compared with NS rats. Consistent with previous studies, the present study demonstrated that high salt diet induced a significant increase in the generation of ROS, sympathoexcitation and blood pressure. Data from this study showed that CoQ10 supplement could attenuate oxidative stress and decrease the sympathetic activity and arterial pressure in high salt-induced hypertension. We may safely draw the conclusion that CoQ10 gives antioxidant function full play in the PVN and plays an important role in the process of blood pressure.

The PVN contains many excitatory and inhibitory neurotransmitters, such as glutamate, NE, and GABA contribute to the pathogenesis of hypertension^36^. NE is the dominant neurotransmitters to excite the sympathetic outflow^37^,^38^, and GABA mediates inhibition of sympathetic activity in the PVN^39^. Glutamate and NE in the PVN are the neurotransmitters that excite the sympathetic outflow, and GABA in the PVN is an important neurotransmitter for regulating inhibition of sympathetic activity^34^. Data from other study showed that CoQ10 may be a promising therapeutic strategy for ameliorating glutamate excitotoxicity in glaucomatous neurodegeneration^14^. Consistent with previous studies, our results demonstrated a lower 67-kDa isoform of glutamate decarbox-ylase (GAD67) expression and higher tyrosine hydroxylase (TH) expression in high salt-induced hypertension, indicating that high salt-diet induced sympathyoexcitation and hypertension may result from the imbalance between the excitatory and inhibitory neurotransmitters in the PVN. The changes of these neurotransmitters were restored after CoQ10 administration. Therefore, CoQ10 supplement was considered to attenuate high salt-induced hypertension by restoring the balance between the excitatory and inhibitory neurotransmitters.

In conclusion, CoQ10 might have potential treatment of hypertension by restoring the balance between excitatory and inhibitory neurotransmitters and the balance between pro- and anti-inflammatory cytokines in the PVN. Our findings provide further evidence and insight of CoQ10 for the prevention and treatment of high salt-induced hypertension, and suggest that CoQ10 may be a promising therapeutic strategy for hypertension. However, further mechanism of interactions between neurotransmitters and inflammatory cytokines within the PVN by CoQ10 is warranted.

## Additional Information

**How to cite this article**: Gao, H.-L. *et al*. Oral CoQ10 attenuates high salt-induced hypertension by restoring neurotransmitters and cytokines in the hypothalamic paraventricular nucleus. *Sci. Rep*. **6**, 30301; doi: 10.1038/srep30301 (2016).

## References

[b1] Zhanatbekova A. K. . Diagnostic and therapeutic strategies for resistant arterial hypertension–focus on countries with emerging economies. Bratislavske lekarske listy 115, 280–286 (2014). 24836409 10.4149/bll_2014_058

[b2] Macmahon S. . Blood-pressure-related disease is a global health priority. Journal of hypertension 26, 2071–2072, doi: 10.1097/HJH.0b013e32830f45d9 (2008). 18806633

[b3] Sacks F. M. . Effects on blood pressure of reduced dietary sodium and the Dietary Approaches to Stop Hypertension (DASH) diet. DASH-Sodium Collaborative Research Group. The New England journal of medicine 344, 3–10, doi: 10.1056/NEJM200101043440101 (2001). 11136953

[b4] Ha S. K. Dietary salt intake and hypertension. Electrolyte & blood pressure : E & BP 12, 7–18, doi: 10.5049/EBP.2014.12.1.7 (2014). 25061468 PMC4105387

[b5] Qi J. . Targeting Interleukin-1 beta to Suppress Sympathoexcitation in Hypothalamic Paraventricular Nucleus in Dahl Salt-Sensitive Hypertensive Rats. Cardiovascular toxicology, doi: 10.1007/s12012-015-9338-7 (2015). 26304161

[b6] Kang Y. M. . Chronic infusion of enalaprilat into hypothalamic paraventricular nucleus attenuates angiotensin II-induced hypertension and cardiac hypertrophy by restoring neurotransmitters and cytokines. Toxicology and applied pharmacology 274, 436–444, doi: 10.1016/j.taap.2013.12.001 (2014). 24342267

[b7] Su Q. . Inhibition of reactive oxygen species in hypothalamic paraventricular nucleus attenuates the renin-angiotensin system and proinflammatory cytokines in hypertension. Toxicol Appl Pharmacol 276, 115–120, doi: 10.1016/j.taap.2014.02.002 (2014). 24576725

[b8] Song X. A. . Inhibition of TNF-alpha in hypothalamic paraventricular nucleus attenuates hypertension and cardiac hypertrophy by inhibiting neurohormonal excitation in spontaneously hypertensive rats. Toxicol Appl Pharmacol 281, 101–108, doi: 10.1016/j.taap.2014.09.004 (2014). 25223692

[b9] Yu X. J. . Interaction between AT1 receptor and NF-kappaB in hypothalamic paraventricular nucleus contributes to oxidative stress and sympathoexcitation by modulating neurotransmitters in heart failure. Cardiovascular toxicology 13, 381–390, doi: 10.1007/s12012-013-9219-x (2013). 23877628

[b10] Sriramula S. . Involvement of tumor necrosis factor-alpha in angiotensin II-mediated effects on salt appetite, hypertension, and cardiac hypertrophy. Hypertension 51, 1345–1351, doi: 10.1161/HYPERTENSIONAHA.107.102152 (2008). 18391105 PMC2736909

[b11] Qi J. . NF-kappaB Blockade in Hypothalamic Paraventricular Nucleus Inhibits High-Salt-Induced Hypertension Through NLRP3 and Caspase-1. Cardiovascular toxicology, doi: 10.1007/s12012-015-9344-9 (2015). 26438340

[b12] Zhang M. . Endogenous hydrogen peroxide in the hypothalamic paraventricular nucleus regulates neurohormonal excitation in high salt-induced hypertension. Toxicology letters 235, 206–215, doi: 10.1016/j.toxlet.2015.04.008 (2015). 25891026

[b13] Tarry-Adkins J. L. . Coenzyme Q10 prevents accelerated cardiac aging in a rat model of poor maternal nutrition and accelerated postnatal growth. Molecular metabolism 2, 480–490, doi: 10.1016/j.molmet.2013.09.004 (2013). 24327963 PMC3854989

[b14] Lee D. . Coenzyme Q10 inhibits glutamate excitotoxicity and oxidative stress-mediated mitochondrial alteration in a mouse model of glaucoma. Investigative ophthalmology & visual science 55, 993–1005, doi: 10.1167/iovs.13-12564 (2014). 24458150 PMC3929080

[b15] Zhou M. . Effects of coenzyme Q10 on myocardial protection during cardiac valve replacement and scavenging free radical activity *in vitro*. The Journal of cardiovascular surgery 40, 355–361 (1999). 10412920

[b16] Burke B. E. . Randomized, double-blind, placebo-controlled trial of coenzyme Q10 in isolated systolic hypertension. Southern medical journal 94, 1112–1117 (2001). 11780680 10.1097/00007611-200111000-00015

[b17] Rosenfeldt F. L. . Coenzyme Q10 in the treatment of hypertension: a meta-analysis of the clinical trials. Journal of human hypertension 21, 297–306, doi: 10.1038/sj.jhh.1002138 (2007). 17287847

[b18] Yamagami T. . Bioenergetics in clinical medicine. Studies on coenzyme Q10 and essential hypertension. Research communications in chemical pathology and pharmacology 11, 273–288 (1975). 1153873

[b19] Ostrowski R. P. Effect of coenzyme Q(10) on biochemical and morphological changes in experimental ischemia in the rat brain. Brain research bulletin 53, 399–407 (2000). 11136995 10.1016/s0361-9230(00)00406-8

[b20] Murad L. B. . Effects of decylubiquinone (coenzyme Q10 analog) supplementation on SHRSP. Biofactors 30, 13–18 (2007). 18198397 10.1002/biof.5520300102

[b21] Yi Q. Y. . Paraventricular Nucleus Infusion of Epigallocatechin-3-O-Gallate Improves Renovascular Hypertension. Cardiovascular toxicology, doi: 10.1007/s12012-015-9335-x (2015). 26162770

[b22] Kang Y. M. . Brain nuclear factor-kappa B activation contributes to neurohumoral excitation in angiotensin II-induced hypertension. Cardiovascular research 82, 503–512, doi: 10.1093/cvr/cvp073 (2009). 19246475 PMC2682616

[b23] Palkovits M. Isolated removal of hypothalamic or other brain nuclei of the rat. Brain research 59, 449–450 (1973). 4747772 10.1016/0006-8993(73)90290-4

[b24] Barber M. . Diabetes-induced neuroendocrine changes in rats: role of brain monoamines, insulin and leptin. Brain research 964, 128–135 (2003). 12573521 10.1016/s0006-8993(02)04091-x

[b25] Miller F. J. . Superoxide production in vascular smooth muscle contributes to oxidative stress and impaired relaxation in atherosclerosis. Circulation research 82, 1298–1305 (1998). 9648726 10.1161/01.res.82.12.1298

[b26] Guggilam A. . Cytokine blockade attenuates sympathoexcitation in heart failure: cross-talk between nNOS, AT-1R and cytokines in the hypothalamic paraventricular nucleus. European journal of heart failure 10, 625–634, doi: 10.1016/j.ejheart.2008.05.004 (2008). 18550427 PMC2593148

[b27] Li H. B. . Central blockade of salusin beta attenuates hypertension and hypothalamic inflammation in spontaneously hypertensive rats. Scientific reports 5, 11162, doi: 10.1038/srep11162 (2015). 26220637 PMC4518230

[b28] Agarwal D. . Chronic exercise modulates RAS components and improves balance between pro- and anti-inflammatory cytokines in the brain of SHR. Basic research in cardiology 106, 1069–1085, doi: 10.1007/s00395-011-0231-7 (2011). 22124756 PMC3261080

[b29] de Wardener H. E. The hypothalamus and hypertension. Physiological reviews 81, 1599–1658 (2001). 11581498 10.1152/physrev.2001.81.4.1599

[b30] Xiang M. . Hemorrhagic shock activation of NLRP3 inflammasome in lung endothelial cells. J Immunol 187, 4809–4817, doi: 10.4049/jimmunol.1102093 (2011). 21940680 PMC3197874

[b31] Cardinale J. P., Sriramula S., Mariappan N., Agarwal D. & Francis J. Angiotensin II-Induced Hypertension Is Modulated by Nuclear Factor-kappa B in the Paraventricular Nucleus. Hypertension 59, 113–U282, doi: 10.1161/Hypertensionaha.111.182154 (2012). 22106405 PMC3268075

[b32] Schmelzer C. . Functions of coenzyme Q(10) in inflammation and gene expression. Biofactors 32, 179–183 (2008). 19096114 10.1002/biof.5520320121

[b33] Abdollahzad H. . Effects of Coenzyme Q10 Supplementation on Inflammatory Cytokines (TNF-alpha, IL-6) and Oxidative Stress in Rheumatoid Arthritis Patients: A Randomized Controlled Trial. Archives of medical research 46, 527–533, doi: 10.1016/j.arcmed.2015.08.006 (2015). 26342738

[b34] Zhang M. . Endogenous hydrogen peroxide in the hypothalamic paraventricular nucleus regulates neurohormonal excitation in high salt-induced hypertension. Toxicology letters 235, 206–215, doi: 10.1016/j.toxlet.2015.04.008 (2015). 25891026

[b35] Yu X. J. . Inhibition of NF-kappa B activity in the hypothalamic paraventricular nucleus attenuates hypertension and cardiac hypertrophy by modulating cytokines and attenuating oxidative stress. Toxicol Appl Pharm 284, 315–322, doi: 10.1016/j.taap.2015.02.023 (2015). 25759242

[b36] Dampney R. A. Functional organization of central pathways regulating the cardiovascular system. Physiological reviews 74, 323–364 (1994). 8171117 10.1152/physrev.1994.74.2.323

[b37] Zhang L. . Effects of Propofol on Excitatory and Inhibitory Amino Acid Neurotransmitter Balance in Rats with Neurogenic Pulmonary Edema Induced by Subarachnoid Hemorrhage. Neurocritical care, doi: 10.1007/s12028-015-0206-x (2015). 26561305

[b38] Jia L. L. . Exercise training attenuates hypertension and cardiac hypertrophy by modulating neurotransmitters and cytokines in hypothalamic paraventricular nucleus. PloS one 9, e85481, doi: 10.1371/journal.pone.0085481 (2014). 24482680 PMC3901693

[b39] Ding L. . GABA in Paraventricular Nucleus Regulates Adipose Afferent Reflex in Rats. PloS one 10, e0136983, doi: 10.1371/journal.pone.0136983 (2015). 26317425 PMC4552845

